# Reproductive suppression, birth defects, and periviable birth

**DOI:** 10.1111/eva.12585

**Published:** 2018-01-10

**Authors:** Ralph Catalano, Tim A. Bruckner, Deborah Karasek, Wei Yang, Gary M. Shaw

**Affiliations:** ^1^ School of Public Health University of California Berkeley CA USA; ^2^ Program in Public Health University of California Irvine CA USA; ^3^ Department of Pediatrics Division of Neonatology Stanford University School of Medicine Stanford CA USA

**Keywords:** birth defects, periviable birth, reproductive suppression

## Abstract

We argue that reproductive suppression has clinical implications beyond its contribution to the burden of spontaneous abortion. We theorize that the incidence of births before the 28th week of gestation, which contribute disproportionately to infant morbidity and mortality, varies over time in part due to reproductive suppression in the form of selection in utero. We further theorize that the prevalence of structural birth defects among survivors to birth from conception cohorts gauges selection in utero. We based these theories on literature positing that natural selection conserved mechanisms that spontaneously abort “risky” pregnancies including those otherwise likely to yield infants with structural birth defects or small‐for‐gestational age males. We test our theory using high‐quality birth defect surveillance data. We identify 479,885 male infants exposed to strong selection defined as membership in conception cohorts ranked in the lowest quartile of odds of a birth defect among live‐born females. We estimate the risk of periviable birth among these infants as a function of selective pressure as well as of mother's race/ethnicity and age. We find that male infants from exposed conception cohorts exhibited 10% lower odds of periviable birth than males from other conception cohorts. Our findings support the argument that selection in utero has implications beyond its contribution to the burden of spontaneous abortion.

## 1 INTRODUCTION

A large literature describes mechanisms that avert or abort gestation in female primates when young conspecifics fail to thrive in prevailing environments (Beehner & Lu, 2013; Wasser & Barash, 1983). With very few exceptions (Coulam, 2016; Quenby, Vince, Farquharson, & Aplin, 2002), the human clinical literature ignores these mechanisms despite their implications not only for the incidence of spontaneous abortion but also for the timing of parturition. We call attention to this gap in the literature by showing that conception cohorts subjected to relatively little selection in utero yield relatively many live births before the 28th week of gestation. Compared to births later in gestation, these “periviable” infants include significantly more small‐for‐gestational age males, suffer greater morbidity, and die much more frequently (Lau, Ambalavanan, Chakraborty, Wingate, & Carlo, 2013). We further argue that these associations may provide clinicians with information useful in anticipating demand for preventive and treatment services.

Half or fewer of human conceptions yield a live birth (Wang et al., 2003; Wilcox, Baird, & Weinberg, 1999). Attrition does not occur randomly from conception to birth. Selection at implantation and early gestation, for example, spontaneously aborts most morphologically, chromosomally, and genetically abnormal fetuses (Coulam, 2016; Teklenburg, Salker, Heijnen, Macklon, & Brosens, 2010). For reasons as yet poorly understood, this early selection appears greater against female than male fetuses (Orzack et al., 2015). After clinical recognition, however, spontaneous abortion appears to discriminate by fetal size and sex, with small male fetuses predominating among those aborted (Mondal, Galloway, Bailey, & Mathews, 2014; Räisänen, Gissler, Saari, Kramer, & Heinonen, 2013). While most of these small male losses occur before the 20th week of gestation, a fraction that varies over conception cohorts occurs later. Important for our purposes, the literature describing periviable birth contends that clinical intervention into what would otherwise have been post‐19th, but pre‐28th, week spontaneous abortions converts a fraction into live births among which small males predominate (Ancel et al., 2015; Chervenak & McCullough, 2013).

Any mechanisms conserved by natural selection to avert maternal investment in less fit offspring would likely include small male fetuses among its targets (Wells, 2000). Male infants receive relatively great maternal investment but suffer greater likelihood of death than any other age by sex group through reproductive age (Bruckner, Helle, Bolund, & Lummaa, 2015; Catalano, Ahern, Bruckner, Anderson, & Saxton, 2009; Lummaa, 2001). This excess mortality among male infants appears for every society and virtually every year for which we have dependable vital statistics (Human Mortality Database, 2017). Among male infants, those born small for gestational age have historically exhibited the greatest risk for infant mortality (Drevenstedt, Crimmins, Vasunilashorn, & Finch, 2008).

The burden of suffering that periviable birth imposes on infants, family, and society compels us to ask why some women spontaneously abort small male fetuses early in gestation while others carry them into and beyond the periviable period. Although representing fewer than 1% of live births in, for example, the United States, periviable infants account for more than 40% of infant deaths (Lau et al., 2013). Among surviving periviable infants, moreover, moderate‐to‐severe morbidity remains elevated well into childhood (Anderson et al., 2016).

Characterizing women who carry small male fetuses past the 19th week of gestation as “loss averse” would seem appropriate given their relative willingness to invest in fetuses that signal low reproductive fitness. Loss‐averse women might implant conceptuses (Coulam, 2016; Teklenburg et al., 2010) or extend the gestation of fetuses (Catalano, Bruckner, Karasek, Adler, & Mortensen, 2016) that other women would, without awareness, reject or spontaneously abort. Where a woman falls on the distribution of loss aversion may reflect either a persistent trait (Haig, 1999; Mishra, Lalumière, & Williams, 2010) or a transient state induced by environmental threats to her well‐being (Catalano, Bruckner, Hartig, & Ong, 2005) or to infant survival (Catalano, Saxton, Gemmill, & Hartig, 2016). The frequency of loss aversion among women contributing to conception cohorts should, therefore, vary from cohort to cohort not only by chance but also due to environmental threats to maternal and infant well‐being.

Variation in the distribution of loss aversion among women contributing to conception cohorts should determine, at least in part, the selective pressure upon fetuses in the cohorts. Conception cohorts with prospective mothers skewed toward loss aversion should exhibit relatively less selection against high‐risk fetuses. Fetuses with structural birth defects that would cause less loss‐averse women to spontaneously abort them might gestate to live birth. Small males in such cohorts might also gestate late into pregnancy before reproductive suppression triggers what would have been, before the advent of modern obstetric practices, a spontaneous abortion or stillbirth but what now becomes a periviable live birth. We, therefore, predict a positive association between structural defects and periviable male births among the survivors to birth of conception cohorts. We determine whether high‐quality birth surveillance data describing 937,597 live births from 222 monthly conception cohorts support this prediction.

## 2 METHODS

We estimate the risk of periviable birth (gestation weeks 20–27) for a male infant as a function of his mother's race/ethnicity and age as well as of the odds of a birth defect among female survivors to birth from his conception cohort. This approach uses the natural variation among conception cohorts in birth defects among females to estimate the average strength of selection on both male and female fetuses in those cohorts. We cannot use the odds of a birth defect among male infants to measure strength of selection because fetuses with birth defects also exhibit elevated risk of early birth (Shaw, Savitz, Nelson, & Thorp, 2001). Using defects among male infants would risk finding a positive association due to nonindependence of birth defects and periviable births among infants of the same sex.

### 2.1 Data and variables

We used birth defect surveillance data collected from 1986 through 2004 in eight California counties (i.e., Fresno, Kern, Kings, Madera, Merced, San Joaquin, Stanislaus, Tulare) that together include numerous structural birth defect phenotypes (corresponding to ICD9 codes 740–758) diagnosed before age 1 from a broad mix of urban and rural populations. These high‐quality surveillance data, described in detail elsewhere (Croen, Shaw, Jensvold, & Harris, 1991), included 937,597 births that we assigned to 222 monthly conception cohorts (i.e., August 1985 through January 2004) based on gestational age at birth. We separated the 479,885 male infants into periviable (i.e., born before the 28th week of gestation) and other births. We excluded multiple births given their high risk for birth defects, spontaneous abortion, and preterm birth.

We characterized male singleton births by maternal race/ethnicity (i.e., non‐Hispanic African American, Asian, Hispanic, and all others) and used non‐Hispanic white as the referent group. We further characterized them by maternal age grouped as 13–19, 20–24, 30–34, and 35 or more years, with age 25–29 as the excluded referent group.

We also characterized male infants by whether their conception cohort experienced strong selection. For the reasons described above, we used the odds of a live‐born female in a cohort exhibiting a birth defect as the indicator of selective pressure. We transformed these odds to their monthly differences (i.e., cohort at month *t* subtracted from that at month *t* + 1) because the raw odds exhibited a downward trend that violates the assumption of a constant mean. The monthly differences vary around a constant mean (i.e., 0) and gauge the degree to which a cohort exhibits higher or lower odds than expected from trend. We identified conception cohorts “exposed” to strong selection as those in the lowest quartile of difference scores. We, therefore, created an exposure variable scored 1 for infants from cohorts with difference scores in the lowest quartile and 0 otherwise.

We added an additional covariate to avoid spurious associations arising from the possibility that periviable birth among male infants and birth defects among female infants share coincident, but not causally induced, autocorrelation including trend, seasonality, regression to the mean, and oscillation (Bressler & Seth, 2011). We specified a variable that expressed the likelihood of a periviable male birth based only on when in the sequence of months in the study the conception occurred. We estimated that variable by applying Box's and Jenkins's well‐established time series modeling routines to the monthly natural logarithm of the odds of a periviable birth among males in the 222 conception cohorts (Box & Jenkins, 1976). This purely empirical approach, recommended for epidemiologic applications (Catalano, Ahern, & Bruckner, 2007), identifies and models autocorrelation in time series and yields expected values that gauge the propensity, conditional on time, of a periviable birth among male survivors to birth from each conception cohort. We assigned the expected conception cohort values back to each live birth and used it as a covariate to control for autocorrelation. This control strategy rules out shared (or opposing) autocorrelation as the source of any association discovered between the odds of a periviable male birth and any independent variable in the logistic regression.

### 2.2 Analyses

We used logistic regression to model the natural logarithm of the odds (i.e., logit) of periviable birth among males as a function of exposure to strong selection in utero adjusting for maternal age, maternal race/ethnicity, and the expected value of a male periviable birth contingent only on time (i.e., propensity in time). We estimated the test equation using SAS, version 9.4.

## 3 RESULTS

The 478,385 male infants in the study included 3,094 born before the 28th week of gestation. Table 1 shows the percentage of all births and periviable births for the maternal age and race/ethnicity categories.

**Table 1:** Sociodemographic characteristics of all male singleton live births and those born before the 28 weeks of gestation, 1986–2004

	All males (*N* = 478,385) *n* (%)	Males born before the 28 wk of gestation (*N* = 3,094) *n* (%)
Maternal race		
Non‐Hispanic white	179552 (37.5)	971 (31.4)
Hispanic white	233321 (48.8)	1488 (48.1)
Black	22156 (4.6)	365 (11.8)
Asian	37553 (7.9)	222 (7.2)
Other	5803 (1.2)	48 (1.6)
Maternal age (mean 25.8)		
13–19	76779 (16.1)	670 (21.7)
20–24	141413 (29.6)	867 (28.0)
25–29	130115 (27.2)	729 (23.6)
30–34	85612 (17.9)	523 (16.9)
35–55	44466 (9.3)	305 (9.9)

Box–Jenkins methods detected seasonality in the logit of male periviable births such that the value observed at month *t* predicted the value at month *t* + 12 better than simply the mean of all values. The best fitting model for the series, therefore, included the mean (i.e., −5.10; SE = 0.03) and an autoregressive parameter at month *t* − 12 (coefficient = −0.14; SE = 0.01).

Table 2 shows the results of estimating the logistic regression model. The odds ratio for membership in conception cohorts exposed to strong selective pressure in utero falls significantly below 1 (i.e., 0.90; 95% Wald Confidence interval 0.82–0.98). The coefficient implies that male infants from conception cohorts in the lowest quartile of odds of a female with birth defects exhibited 10% lower odds of periviable birth than males from other cohorts. The fact that cohorts with unusually low odds of birth defects among females also exhibited unexpectedly low likelihood of male periviable births further implies, as argued above, that “risky” structural defects and sex‐specific impediments to fetal growth appear early in gestation and that risk‐averse mothers spontaneously abort pregnancies when detecting signals of either.

**Table 2:** Coefficients for test equation predicting the likelihood of periviable birth among males from propensity in time, exposure to selective pressure in utero, maternal age, and maternal race/ethnicity

Predictor	Point estimate	Wald lower 95% bound	Wald upper 95% bound
Propensity in time	2.56	1.17	5.61
Exposed to selective pressure	0.90	0.82	0.98
Age 13–19 (vs. 25–29)	1.47	1.33	1.64
Age 20–24 (vs. 25–29)	1.06	0.96	1.18
Age 30–34 (vs. 25–29)	1.10	0.98	1.23
Age 35–55 (vs. 25–29)	1.23	1.08	1.41
Hispanic (vs. white)	1.15	1.06	1.25
African American (vs. white)	2.96	2.62	3.34
Asian (vs. white)	1.07	0.93	1.24
Other race (vs. White)	1.50	1.12	2.00

The coefficient for propensity for periviable birth contingent on time exceeded 1 (i.e., 2.56; 95% Wald Confidence Interval 1.17–5.60). Seasonality detected by the Box–Jenkins modeling, described above, of the odds of periviable birth among males implies this association. The fact that the association remains significant in the test model implies that seasonality in the likelihood of a male periviable birth does not arise from seasonality in the other predictors in the equation.

Males born to non‐Hispanic white mothers exhibit the lowest risk of periviable birth while those born to African American mothers exhibit the highest risk (i.e., 2.96; 95% Wald Confidence Interval 2.62–3.34). Epidemiologic literature describing periviable birth in the United States predicts this association (DeFranco, Hall, & Muglia, 2016).

We repeated our analyses for periviable births among females. The arguments summarized at the outset imply that we should find no association or one smaller than that for males because small daughters do not represent as great a risk to maternal fitness as do small sons. We applied all the analytic steps described above but to the 2,570 periviable infants among 456,306 live female births. We found no association (OR = 1.01; 95% Wald Confidence Interval 0.92–1.11).

The fraction of pregnant women informed by prenatal screening increased during our test period. The State of California made such screening available by law in 1986. Approximately 65% of infants born in the State had been screened in utero during our test period (Catalano et al., 2012). Elective abortion, therefore, may have also varied not only because maternal risk aversion changed but also because information about fetuses changed. This circumstance, in turn, could have changed the population at risk of periviable birth via a mechanism other than spontaneous abortion. We attempted to estimate the degree to which this circumstance could have affected our results. We assigned to each male birth the log of the odds of a female periviable birth in his conception cohort. This variable plausibly adjusts the odds of male periviable birth for phenomena, including elective abortion, that affect fetal loss in both sexes but not for the male‐specific mechanisms assumed by our theory. The results of this augmented test did not differ from those of our primary test in that the risk ratio (i.e., 0.90) for our exposure variable, and its Wald 95% confidence interval (i.e., 0.82–0.98) did not change.

## 4 DISCUSSION

Our findings support the argument that selection in utero has clinical implications beyond its contribution to the burden of spontaneous abortion. We show that conception cohorts likely subjected to relatively strong selective pressure in utero exhibit relatively low frequency of periviable male births. Our results support the theory of “strategic parturition” that argues that signals of fetal fitness detected early in gestation affect the mechanisms that regulate, at least in part, the timing of parturition (Catalano et al., 2016; Haig, 1999).

We attribute variation among conception cohorts in their rates of periviable birth to variation in loss aversion among women who conceived the cohorts. We acknowledge that invoking the construct of gestational loss aversion, despite its intuitive appeal, raises issues we cannot address with our data. Other literature (Karasek et al., 2015) has, for example, asked whether women who exhibit gestational loss aversion also exhibit loss aversion in investment choices they can describe making. Research suggests they may because the male twin ratio of birth cohorts, which measures the spontaneous abortion of the smaller of male twin fetuses, falls in Sweden when households report rising economic risk aversion (Karasek et al., 2015). The literature also reports that Danish conception cohorts that yield more than expected spontaneous abortions also yield more than expected elective abortion of gestations with no clinical indication of fetal defect (Catalano, Bruckner, et al., 2016). If cognitively accessible and inaccessible decisions share preferences and biases, inexpensive and noninvasive assessments devised by economists to measure loss aversion in individuals may provide information that improves algorithms for identifying women at risk of periviable birth (Choi, Kariv, Müller, & Silverman, 2011).

Governments (United States of America National Institutes of Health, 2015) and obstetric care providers (Raju, Mercer, Burchfield, & Joseph, 2014) have called for research into the ethical, clinical, and public health implications of advancements in obstetric practices that save risky gestations at the threshold of viability. Our research complements these clinically focused efforts by demonstrating that characteristics of conception cohorts—rather than those of gestations at or near delivery—predict the likelihood of periviable birth.

Strengths of our study include longitudinal observation of a large population and nearly exhaustive (i.e., 95%) ascertainment of the periviable births and of birth defects. This coverage permits stable estimation of population rates for relatively rare events and provides sufficient statistical power to detect small but important associations. Assignment of more than 470,000 males to conception cohorts improves upon previous work that relied on date of live birth to infer temporal ordering of selection processes during pregnancy (Bruckner, Catalano, & Ahern, 2010).

Limitations of our test include that low counts of specific birth defect types in any month precluded identifying which defects best signal loss aversion. We do not, moreover, know how widely our results may apply despite that our test counties include urban, rural, and demographically as well as socioeconomically diverse communities.

The literature includes much speculation concerning the evolutionary origins of decisional biases and preferences (Zhang, Brennan, & Lo, 2014) but few empirical tests of how these phenomena affect reproduction. Such tests should, in the future, prospectively monitor cohorts of women who, based on a combination of biomarkers and behavioral assessments, appear most likely to exhibit unusually high or loss aversion during pregnancy. We anticipate that development of such diagnostic tools for women of childbearing age may not only improve our understanding of selection in utero but also contribute to the management of gestations at risk of periviable birth.

## DATA ARCHIVING STATEMENT

Data used in these analyses contain protected health information and therefore require human subjects’ approval from the State of California before dissemination and analysis. Please contact Gary M Shaw (gmshaw@stanford.edu) if interested in obtaining the data and code for replication.
